# Dogs are more pessimistic if their owners use two or more aversive training methods

**DOI:** 10.1038/s41598-021-97743-0

**Published:** 2021-09-24

**Authors:** Rachel A. Casey, Maria Naj-Oleari, Sarah Campbell, Michael Mendl, Emily J. Blackwell

**Affiliations:** 1 Dogs Trust, Canine Behaviour and Research, Clarissa Baldwin House, 17, Wakley Street, London, UK; 2 Gornate Olona, Italy; 3 Vets for Pets, 350 Southchurch Drive, Clifton, Nottingham, UK; 4 Bristol Veterinary School, University of Bristol, Bristol, UK

**Keywords:** Emotion, Animal behaviour

## Abstract

Domestic dogs are trained using a range of different methods, broadly categorised as reward based (positive reinforcement/negative punishment) and aversive based (positive punishment/negative reinforcement). Previous research has suggested associations between use of positive punishment-based techniques and undesired behaviours, but there is little research investigating the relative welfare consequences of these different approaches. This study used a judgement bias task to compare the underlying mood state of dogs whose owners reported using two or more positive punishment/negative reinforcement based techniques, with those trained using only positive reinforcement/negative punishment in a matched pair study design. Dogs were trained to discriminate between rewarded and unrewarded locations equidistant from a start box, and mean latencies recorded. Their subsequent latency to intermediate ‘ambiguous’ locations was recorded as an indication of whether these were perceived as likely to contain food or not. Dogs trained using aversive methods were slower to all ambiguous locations. This difference was significant for latency to the middle (Wilcoxon Z = − 2.380, P = 0.017), and near positive (Wilcoxon Z = − 2.447, P = 0.014) locations, suggesting that dogs trained using coercive methods may have a more negative mood state, and hence that there are welfare implications of training dogs using such methods.

## Introduction

In contrast to commercially reared livestock, the welfare implications of different husbandry approaches for domestic dogs have received relatively little research. There is a common perception that because dogs live in domestic settings with their owners they have a ‘good life’. However, life experiences for dogs are potentially very variable because they largely depend on the circumstances, knowledge, attitudes and lifestyle of their owners^1–3^. One aspect in which owners vary considerably is in the choice of training methods used to teach new behaviours, or to alter undesired behaviours, in their dogs. Approaches to dog training vary from the use of rewards (such as attention, praise, play or food) when dogs show a desired response, to the use of aversive interventions when they display unwanted behaviour^4,5^.

The different approaches are often described by dog training practitioners using terms developed by B.F. Skinner in the 1930’s^6^. These terms describe the increase (reinforcement) or decrease (punishment) in the likelihood of a specific target behaviour given the (positive) application or (negative) withdrawal of different stimuli. ‘Positive punishment’ is defined as the probability of a behaviour occurring later being *decreased* on the *application* of a stimulus; ‘negative reinforcement’ is defined as the probability of a behaviour occurring later being *increased* on *withdrawal* of a stimulus; ‘positive reinforcement’, is defined as the probability of a behaviour occurring later being *increased* with the *application* of a stimulus; and ‘negative punishment’ is defined as the probability of a behaviour being *decreased* with the *removal* of a stimulus. However, positive punishment and negative reinforcement inevitably occur together depending on the focal behaviour described by the these definitions: as one behaviour within a context increases, another will be decreased. For example, the action of spraying a dog with water may both reduce (positively punish) jumping up, but also increase (negatively reinforce) standing on all four paws when the spraying is stopped. Similarly, a dog could be positively reinforced with attention for sitting to greet people (behaviour increasing with the intervention), and negatively punished for jumping up by withdrawal of attention if the dog does not sit^7^. Because these two categories occur together within contexts, we have combined training techniques used by owners into ‘reward based’ (both positive reinforcement and negative punishment) and ‘aversion based’ (both positive punishment and negative reinforcement).

Many veterinary, clinical behaviour, dog training and animal welfare organisations promote the use of reward based training methods for dogs. Arguments for this recommendation are listed below:

1. That many undesired behaviours arise because dogs are anxious or fearful, and the implementation of more aversive methods may enhance negative emotional states, resulting in further problems including aggression^8–10^.
2. That dogs may associate the application of an aversive stimulus with unintended and coincidental events^9^. For example, an electric stimulus used to deter a dog from running away may be applied when the dog is approaching a child, with the risk of the aversive event becoming associated with children.
3. That the use of aversive methods may inhibit behaviours in that context in which the punishment is applied, but not alter underlying emotional state, potentially leading to the subsequent return of the problem behaviour or alternative responses on exposure to the precipitating cue in other contexts.
4. That the use of aversive methods can create confusion and frustration in dogs because punishment of an undesired behaviour alone does not enable the dog to understand what is required as the appropriate response to a cue.
5. That aversive methods can risk causing physical injury or pain to dogs^9^.

Previous studies have suggested associations between the use of more aversive training methods with unwanted behaviours^4,5,8^, and an increased occurrence of aggressive behaviour where such techniques are used^1,8,11^. However, the causality of these relationships is unclear: whilst it may be that use of approaches involving aversive stimuli increase the risk of fear and aggression responses, it is also possible that owners whose dogs show such behaviours are more likely to resort to these types of techniques.

There are also some indications that the use of more aversive methods of training have direct impacts on the welfare of dogs. For example, German Shepherds trained with electronic (“shock”) collars were shown to display more behavioural signs of fear towards their handlers than those trained with other methods^12^, and dogs showed increases in cortisol when handlers tended to use discipline during interactions as compared to engaging in more affiliative play^13^. However, there are often difficulties in interpreting behavioural and physiological indicators of welfare, particularly when single indicators are used. Measures can be influenced by other factors and may not necessarily be associated with emotional states of particular valence^14^.

A relatively novel approach to welfare assessment is the measurement of ‘cognitive bias’^15^. Cognitive biases are observed where affective state influences human or animal responses to environmental stimuli, through differences in how past events are remembered (‘memory bias’), ambiguous or future events are evaluated (‘judgement bias’), or attention is focused (‘attention bias’)^15^. Moods are defined as relatively enduring affective states which are detached from immediate triggering stimuli^16,17^. However, mood states are thought to be influenced by cumulative experience, such that individuals which experience repeated aversive events, or fail to achieve expected rewards, will have a more negative mood state than those successfully achieving rewards and avoiding threats^17,18^. These changes in mood state with environmental circumstances are considered adaptive as they enable individuals to adjust responses to ambiguity and expectations of future events according to previous achievement (or not) of rewards, and avoidance (or not) of aversive stimuli^16,17,19^. For example, individuals experiencing repeated aversive stimuli and hence in a negative mood are predicted to show a more cautious, negative interpretation of future or ambiguous events. The judgement bias test developed by Harding^20^ aims to evaluate whether animals have positive or negative expectations in ambiguous situations and hence to use these judgements as proxy indicators of, respectively, positive or negative affect or mood^16,21^.

Previous animal studies have confirmed that measures of judgement bias change with manipulations of the environment likely to influence mood states, such as the removal or addition of enrichment^22–26^, and judgement bias methods developed for non-human animals^20,27^ have been successfully adapted for dogs^18^. Given that mood is hypothesized to be influenced by experience of reward and aversion, this approach is appropriate to evaluate differences in affective state between dogs trained using rewarding or aversive stimuli. Previous research has investigated differences in cognitive indicators of affect and welfare between groups of dogs attending training classes where different approaches to training were used^28^. Dogs attending classes advocating mainly aversive training methods were found to be more ‘pessimistic’ in a cognitive bias task than dogs from training classes adopting a predominantly reward-based approach. The aim of this project was to further examine this relationship by using a matched pair study design to test the hypothesis that dogs trained using owner reported training techniques based on aversion (i.e. using positive punishment/negative reinforcement^7^) are more likely to show a relatively ‘pessimistic’ judgement bias as compared to a breed, age and sex matched control population in which these techniques had not been used.

## Results

**Figure 1:**
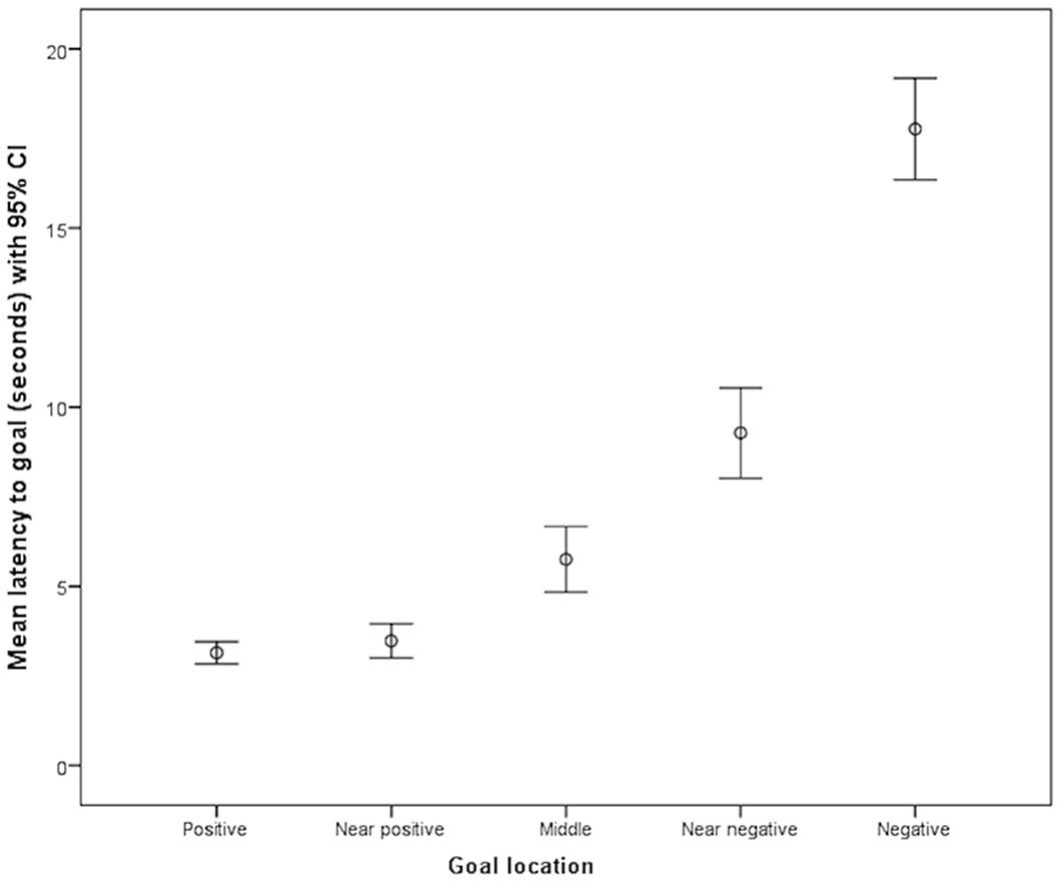
Mean latency to positive and negative locations and unadjusted latencies to probe trials, showing 95% confidence intervals, during the test phase for all dogs.

A spatial judgement bias task was used^18,27^ in which dogs were trained that a bowl would contain reward (food) on trials in which it was placed at a standardized (positive) location on the left side of a test arena, and no reward on trials in which it was placed at a standardized (negative) location on the right side of the arena (reward-location combinations remained the same for each dog and were counter-balanced across dogs). Once dogs had learnt this spatial discrimination and were running faster to the bowl on trials in which it was on the rewarded side, trials in which the bowl was placed in one of three ambiguous locations (e.g. half-way between the left and right locations) were presented. Latency to move to the bowl in these trials was used as an indicator of whether the dog anticipated food to be present (short latency—positive (‘optimistic’) interpretation of ambiguity inferred to reflect relatively positive affect) or absent (longer latency—negative (‘pessimistic’) interpretation of ambiguity inferred to indicate a relatively negative affective state). 113 tested dogs completed the cognitive bias task. Of these 13 were removed from the dataset because suitable matches were not identified for testing, leaving 100 subjects for analysis: 50 trained with two or more aversive methods (AT group), and 50 who had not experienced these training methods according to owner report (RT group). A breakdown of the breeds, age categories, sex and neuter status for these dogs are show in Table 1. The mean latencies to reach goal locations in the test phase for all dogs are shown in Fig. 1.

**Table 1:** Signalment information of tested subjects in both groups.

Matching characteristic	Category	Number in each group (AT and RT)
Breed type	Cross breed	7
	Border collie	5
	German shepherd	2
	Labrador retriever	6
	Golden retriever	2
	Cocker spaniel	3
	Springer spaniel	8
	Flat-coated retriever	1
	Irish setter	1
	Bassett hound	1
	Beagle	1
	Border terrier	3
	West highland white terrier	1
	Jack Russell terrier	7
	Boxer	2
Sex and neuter status of dog	Male entire	5
	Female entire	8
	Male neutered	19
	Female neutered	18
Age category	6 < 18 months	8
	18 < 60 months	29
	> 60 months	13

No significant effect of rewarded bowl location (left or right) was found for latencies to probe goal locations, suggesting that side biases were unlikely to have influenced results. A final trial in which the bowl was presented in the negative location but contained food was used to investigate whether dogs might be using olfactory cues in the task. In fact, latency to the bowl location on this trial tended to be even slower than mean latencies to this location during the test phase (Wilcoxon Z = − 2.721, P = 0.07) indicating that responses were unlikely to have been influenced by olfactory cues.

Dogs in the aversion training (AT) group and the reward training (RT) group did not differ with respect to the number of trials to reach criterion (Wilcoxon, Z = − 0.368, P = 0.713) suggesting that the groups did not vary in their ability to learn the spatial discrimination task. There was also no significant difference between groups in the mean latency to the negative location or the mean latency to the positive location during the test phase (Wilcoxon Z = − 1.105, P = 0.836 and Z = − 1.105, P = 0.269 respectively).

**Figure 2:**
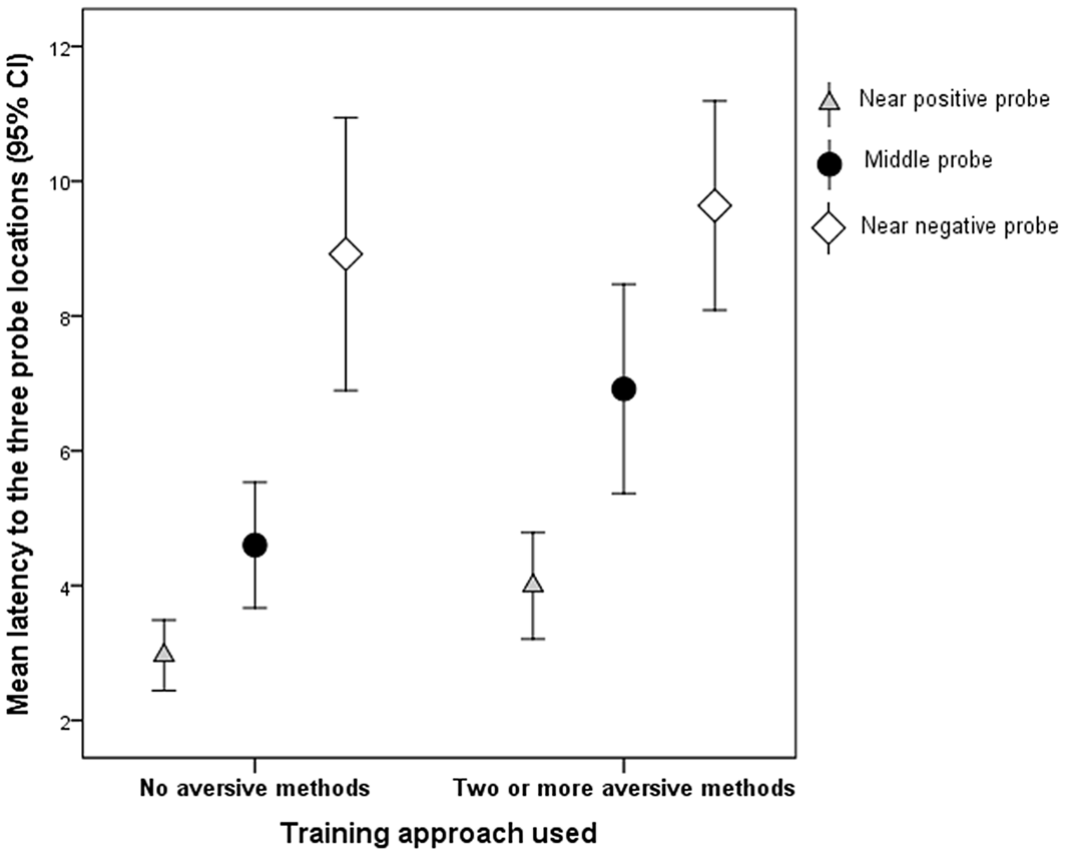
Mean latency for each intermediate ‘probe’ goal location, divided by training group.

Dogs trained using no aversive methods (reward training; RT) had a lower latency (i.e. ran faster) to all probe goal locations than those trained using two or more aversive methods (aversion training group: AT; Fig. 2). This difference was significant for latency to the middle probe location (Wilcoxon Z = − 2.380, P = 0.017), and for latency to the near positive probe location (Wilcoxon Z = − 2.447, P = 0.014).

## Discussion

This study used a matched pair study design to compare dogs whose owners reported using at least two training methods involving the application of an aversive stimulus with those trained with rewards only. Because mood states are thought to be influenced by cumulative experience^16,17^, we hypothesized that dogs who experienced aversive techniques during training would have a more negative mood state than those whose training was focused on rewarding desired behaviours. Measurement of latency to reach ambiguous bowl locations in the judgement bias task supported this hypothesis, with dogs in the group trained with aversive methods being more ‘pessimistic’ than those trained without these techniques, and moving significantly more slowly to the bowl when on trials when it was in the central ambiguous location or that nearest the trained rewarded location.

This ‘pessimistic’ bias indicates that dogs which have been exposed to more aversive training methods have a decreased expectation of reward than those which have not experienced these methods. This may reflect a negatively biased processing of ambiguous information, as is found in depressed people^29,30^ rats exposed to chronic psychosocial stress^31^, and in a variety of other judgement bias studies involving animals in putative negative affective states^25,26^. The results support the findings of Vieira de Castro et al.^28^, suggesting that the use of aversive dog training methods may induce longer-term negative mood states and hence poorer welfare compared to training methods that do not involve aversion.

It is important to note, however, that the relationship identified is correlational rather than causal. It is possible that rather than aversive training methods generating a negative affective state which leads to pessimistic decision-making under ambiguity, dogs may show stable individual differences in the way in which they interpret situations^32,33^ that influence their owners’ use of aversion based methods of training. For example, dogs with a more pessimistic cognitive bias may be more likely to develop fear associated behaviour problems which owners find difficult to manage, resulting in greater use of punitive techniques. Further research is needed to fully understand the causal direction of the observed relationship between experience of reward and aversion and cognitive bias.

Some caution is required in interpreting these findings as groups were defined according to owner reported use of training methods. Although this was checked on the telephone on booking appointments, and verbally with owner at time of the visit, there was no adjustment for how often the target methods were used, nor how long ago they were last used –factors which may have made the experience of aversive training methods variable in the AT group. However, the risk of classifying dogs in the AT group who had only experienced such methods minimally was reduced by having an inclusion criterion of owners using at least two of the listed methods. Such owners were assumed to be more likely to have an overall ethos of using this type of training method. There is also potential for variation in how these methods were applied to dogs. For example, application of a punisher poorly contingent with the behaviour of the dog is likely to be more stressful for the animal than where it is appropriately applied^12^. Nevertheless, our findings appear to replicate those of Vieira de Castro et al.^28^, where categorization of groups was conducted based on attendance to different training classes, with methods of training further categorized through video analysis of sessions.

The other limitation was that the inclusion criteria for the reward trained group was that owners had not used the specific methods listed in Table 2. These owners may, however, have used other aversive methods that were not specified. In addition, dogs in both groups may have experienced different training methods in the past (e.g. if they had been rehomed). Finally, owners in both groups reported using reward based methods to train their dogs (e.g. praise, play and food treats) as it is difficult to find owners who do not use any such methods with their dog. This means that the groups were defined according to whether owners *also* reported using aversive methods—a comparison between those using only positive reward to those using both positive reward based and positive punishment based methods. It is therefore possible that group differences may be influenced by the relative consistency of methods used by owners, as also suggested by Hiby^5^.

**Table 2:** Dogs were included in the aversive training group if owners reported using two or more of these methods, and included in the reward training group if the owner reported never having used any.

Training method
Bark activated citronella collar
Remote activated citronella collar
Pet corrector
Physical punishment (e.g. smacking or shaking)
Remote activated electronic collar
Bark activated electronic collar
Water pistol
Check or choke chain
‘Rattle can’ or other sound based ‘distraction’ method

It is possible that variables such as breed, gender, neuter status and age may influence cognitive bias. Matching pairs of dogs by these criteria in this study enabled us to conclude with greater confidence that methods used in training rather than demographic variables were associated with mood state, and hence welfare, in dogs. The strength of this study design adds weight to the previous findings of Vieira de Castro et al.^28^. Further research to investigate the extent to which cognitive biases do or do not vary with factors such as breed, individual personality, learning or stress coping style of dogs or owner based factors such as attitude or lifestyle would be valuable, allowing future studies to better control for any confounding factors and also to be less constrained in case recruitment for those characteristics found to be unrelated to judgement biases.

This study may have some general implications for animal training methods, including in other species. For example, in line with our findings, training animals to perform desired responses using only positive reinforcement for procedures in laboratory and zoo situations has been shown to have considerable benefits for welfare^34,35^. Individual variation in sensitivity to rewarding and aversive stimuli may influence the appropriateness of different training approaches. For example, just as human patients with clinical depression have been shown to have a hypersensitive response to punishment^30^, more ‘pessimistically’ biased dogs may be more sensitive to application of punishment, and perhaps require less punitive methods in training than more ‘optimistic’ or less punishment sensitive individuals. However, given that depressed people also have a reduced sensitivity to reward^36^, it may be that reward-based training alone is not sufficient to alter behaviour in animals with a more ‘pessimistic’ bias, although Beevers et al.^37^ suggest that punishment sensitivity is a more important component of depression than reward hyposensitivity. Further research is required to assess the relevance of differences in sensitivity to reward and punishment on dog behaviour, learning and welfare. Inferences from this study are limited by the fact that the cognitive bias test used did not, for ethical reasons, involve any aversive stimulus (just lack of reward).

In conclusion, the results of this study have highlighted further the importance of understanding potential welfare implications associated with training methods used in domestic dogs. The study replicates and strengthens the important findings of Vieira de Castro et al.^28^ through use of a matched pair study design to reduce the impact of confounding variables and using an increased sample size. We demonstrated that dogs appear to show a more pessimistic cognitive bias where trainers report using two or more aversive training methods, and this has important implications for the kind of advice given to dog owners by veterinary surgeons, breeders, behaviourists or trainers, particularly for new owners or those acquiring a puppy. Although alternative interpretations should be considered as discussed above, because of the potential welfare implications of using two or more punishment-based techniques identified in this paper, we believe that professionals should advise owners to use the least aversive methods available to them to modify their dog’s behaviour.

## Materials and methods

### Subject recruitment and matching

Dog owners living in the south-west of the U.K. were recruited to take part in the study using a number of approaches: those who had indicated willingness to take part in the further research as part of previous questionnaire studies^38,39^; through veterinary practices, dog training clubs, forums, breeder lists and breed societies. Owners responding to recruitment calls were contacted initially by telephone or e-mail, and subjects were selected to take part in the study if they fulfilled the following inclusion criteria:

- Owners were willing for researchers to visit their house on a single occasion for a period of up to 3 h.
- Owners were willing to complete a questionnaire and sign an informed consent form.
- Owners had an area on their property of sufficient size to set out a 3 m × 4 m unobstructed arena for conducting the judgement bias task.
- Dogs were between 6 months and 12 years of age.
- Dogs were healthy, sound, and able to eat normal commercial dog food.
- Entire females were not in season at the time of testing.
- Dogs were not on any medication likely to influence their behavior.
- Dogs did not show human-directed aggression, for safety during testing.
- Dogs had *either* been regularly trained using *two or more* of the methods listed in Table 2 (Aversion-based training (AT) group), *or* had been trained with *none* of the methods listed in Table 2 (Reward-based training (RT) group) (see Table 2).

Only one dog from each household was selected for inclusion in the study. Initially all recruited dogs fulfilling these criteria were tested, but as recruitment progressed, dogs were additionally selected based on matching pairs of cases between the two groups. Criteria by which dogs were pair matched between groups were: specific breed; age category (6 months to < 18 months; 18 to < 60 months; over 60 months); sex and neuter status (male entire; male neutered; female entire; female neutered). Appointments were made with recruited owners by telephone, and each was sent information about the study plus a participation consent form prior to visits. They were also asked to complete a short questionnaire to verify their previous responses regarding training methods used with the focal dog. All owners gave informed consent for the use of their dog and data in the study. Each owner and dog was visited by a pair of researchers on a single occasion.

### Judgement bias test

**Figure 3:**
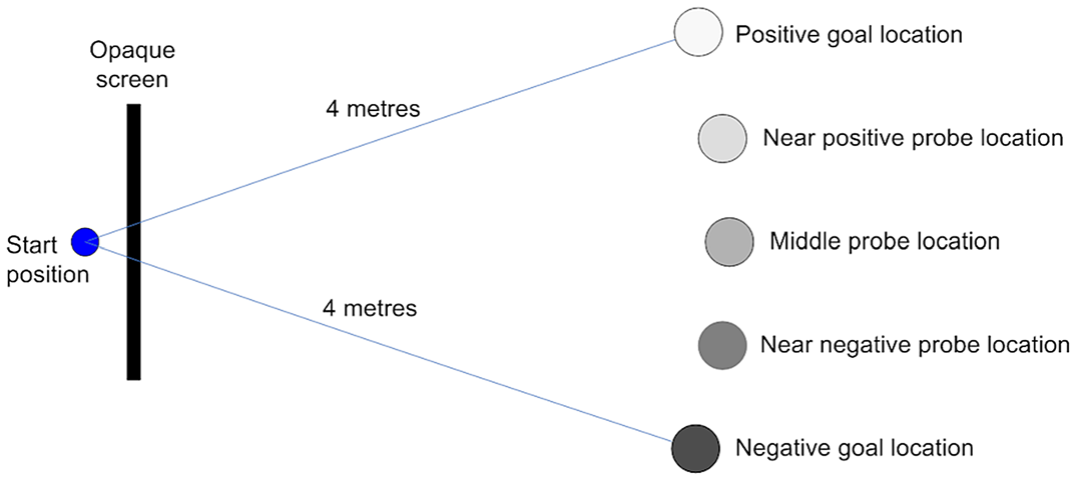
Experimental set-up for measurement of judgement bias in dogs.

Each dog was tested for judgement bias during the visit, using methodology described fully in Mendl et al.^18^, and summarized here. A 4 m × 3 m area was measured and set up as shown in Fig. 3. Masking tape was used to indicate the positions for placement of food bowls during training and testing. The dog was positioned at the start location, behind an opaque barrier, before each training and test trial. During the training phase, dogs were taught to discriminate between a single food bowl placed either on the right or left side of the arena through rewarding approach to one location with a small amount of food and not the other. The rewarded (Positive; P) and unrewarded (Negative; N) sides were randomly allocated to left or right between dogs but stayed consistent within subjects. Cues other than spatial location were controlled for by making the same preparatory noises in ‘baiting’ each bowl and adding but removing food from the negative side to reduce any potential difference in olfactory cues. The time taken for dogs to reach each goal from the starting position was measured in seconds. Dogs were given up to 30 s to reach the target—where they did not reach the goal location, they were given a maximum latency score of 30 s. After each trial, dogs were returned behind the screen whilst the bowl was baited for the next trial. Positive and Negative trials were presented in a pseudo-random order, such that each side was not presented more than twice consecutively, to reduce the risk of side biases developing.

The criterion for moving to the test phase was all latencies to P less than all latencies to N for six consecutive trials. For the testing phase, dogs were presented with three unrewarded ‘probe’ trials with bowls placed in ambiguous locations, spaced at equal quartiles between the positive and negative locations (near positive (NP), middle (M) and near negative (NN); Fig. 3). The probe locations were presented in in the following order: M, NP, NN, NP, NN, M, NN, M, NP (each location was presented first, second or third in each block of three test trials). The nine test trials were each separated by four training trials. These training trials were also pseudo-randomly ordered, ensuring two negative and two positive trials in each block to avoid extinction of the response. The latency to each probe and training trial was recorded. Greater latencies in probe trials were interpreted as the dog judging the bowl to be less likely to contain food (and hence a more ‘pessimistic’ cognitive bias), and faster speeds a greater anticipation of food (and hence a more ‘optimistic’ cognitive bias).

At the end of testing, dogs were presented with a bowl containing food at the negative goal location and the latency recorded. This was to eliminate the possibility that dogs were responding in trials based on direct interpretation of olfactory cues, rather than expectation of reward. Methods were carried out in accordance with relevant guidelines and regulations, and the methodology was approved by the local University of Bristol Ethical Review Board (UIN UB/08/003) for both human and dog participants.

### Statistical analysis

Any tested subjects without pair-matched controls were removed from the dataset. Mean latencies to reach the P and N locations presented during the test phase (N = 32 presentations) were calculated, as were mean latencies to each of the three probe trials (NP, M and NN; 3 presentations of each). Latencies to probe goal locations, number of trials to reach criterion, and latency to reach the final rewarded presentation at the negative goal location were all tested for normality. None were normally distributed even after transformation hence non-parametric tests were used for all analyses.

Latencies to each probe were compared between dogs where the positive goal was located on the left and those where it was on the right, to check for side biases, using a Mann–Whitney *U* test. The latency to the final rewarded bowl in the negative location was compared with the mean latency to the negative goal during the test phase, to check for the potential influence of olfactory cues on responding, using a Wilcoxon signed rank test. The number of trials to reach criterion for testing was compared between AT and RT groups to test for differences in ability to learn the spatial discrimination task with a Wilcoxon matched pairs test. The AT and RT groups were also compared for mean latency to each probe goal location using Wilcoxon matched pairs tests. All analyses were carried out using IBM SPSS Statistics v19. An open access datafile is available at: 10.5061/dryad.312s0.

## Data Availability

The datasets generated during and/or analysed during the current study are available from the corresponding author on reasonable request.
